# Isolation and Characterization of Extracellular Vesicles From *Ascochyta rabiei*, a Necrotrophic Fungal Chickpea Pathogen

**DOI:** 10.1002/pmic.70060

**Published:** 2025-10-20

**Authors:** Matin Ghaheri, Chamindie Punyadeera, Ido Bar, Prabhakaran T. Sambasivam, Abolfazl Jangholi, Donovan Garcia‐Ceron, Muhammad J. A. Shiddiky, Rebecca Ford

**Affiliations:** ^1^ School of Environment and Science Griffith University Queensland Australia; ^2^ Institute For Biomedicine and Glycomics (IBG) Griffith University Queensland Australia; ^3^ Department of Ecological, Plant and Animal Science, La Trobe Institute of Sustainable Agriculture and Food La Trobe University Bundoora Victoria Australia; ^4^ Rural Health Research Institute Charles Sturt University Orange New South Wales Australia

**Keywords:** *Ascochyta rabiei*, chickpea, extracellular vesicle (EV), fungi, plant‐pathogen interaction, proteomics

## Abstract

Extracellular vesicles produced by pathogenic organisms play a role in host recognition and invasion across kingdoms, including fungi interacting with their plant hosts. Little is known about EVs from phytopathogenic fungi compared to human fungal pathogens. *Ascochyta rabiei* is an endemic and widespread necrotrophic ascomycete fungus that causes a significant impact on chickpea production. The presence of EVs generated by *A. rabiei* during the invasion of chickpea has not previously been reported. Here, *A. rabiei* EVs were identified and isolated from liquid fungal broth cultures, both with and without host, using optimized ultracentrifugation and filtration techniques. Nanoparticle tracking analysis, transmission electron microscopy, and mass spectrometry revealed 40–200 nm cup‐shaped, double‐membraned structures. Protein cargo analysis showed significant variations depending on host presence, affecting EV production, size, and composition. The cargo included proteins involved in vesicle transport, basic cellular and oxidation/reduction‐related processes, as well as pathogenesis. Some were upregulated in the presence of the host, likely facilitating immune modulation, plant defence alteration, or pathogen establishment. These findings highlight the presence and functional importance of *A. rabiei* EVs in the interaction with and invasion of chickpea. Future research should concentrate on characterizing EVs under various growth circumstances and confirming fungal‐specific EV markers.

**Summary:**

- This study presents the first characterization of extracellular vesicles (EVs) produced by *Ascochyta rabiei*, a major phytopathogenic fungus affecting chickpea.
- An optimized ultracentrifugation and filtration‐based isolation method enabled efficient EV recovery without reliance on specialized equipment. Comprehensive proteomic analysis revealed that EV production, size distribution, and protein composition were modulated by the presence of the host.
- Identified EV‐associated proteins were linked to virulence, oxidative stress response, cell wall modification, and effector functions, suggesting a role in host‐pathogen interactions.
- These findings advance the current understanding of fungal EVs in plant infection processes and establish a foundation for future studies investigating their functional significance in pathogenesis.

**Abbreviations**
*A. rabiei*
: *Ascochyta rabiei*
EV: extracellular vesicle
LFQ: label‐free quantitation
Log2(FC): log2(fold‐change)
NTA: nanoparticle tracking analysis
PDB: potato dextrose broth
TEM: transmission electron microscopy.

## 1 Introduction

Extracellular vesicles (EVs) are lipid bilayer‐delimited particles that are naturally released by both eukaryotic and prokaryotic cells under normal and pathophysiological circumstances [1, 2, 3]. Phenotypically, fungal EVs vary in size, ranging from around 20 to 1000 nm in diameter [4], with spherical, cup‐shaped, or rounded structures [5, 6], and carry cargoes comprising a range of biomolecules, including proteins, nucleic acids (such as DNA, RNA, and microRNAs), lipids, and metabolites [7, 8, 9]. However, the cargo varies depending on the physiological state of the organism, including when derived from a pathogen and in the presence of a host species [10]. EVs have been isolated from filamentous and yeast pathogenic fungal species, the majority of which infect humans, but with a small number known to cause plant disease. To date, these are *Botrytis cinerea* [11, 12], *Fusarium oxysporum* f. sp. *vasinfectum* [7, 13], *Fusarium graminearum* [14], *Zymoseptoria tritici* [15], *Ustilago maydis* [16], *Colletotrichum higginsianum* [17], *Penicillium digitatum* [18], *Phytophthora capsici* [5], and *Alternaria infectoria* [19].

Accordingly, EVs have been reported to have multiple functions within pathogenic fungal‐plant interactions. Fungal EVs facilitate the penetration of fungus into plant tissues and nutrient uptake as they contain cell wall‐degrading enzymes [14, 17]. For example, *F. oxysporum* EVs were reported to contain cell wall‐degrading enzymes such as chitinases and glucanases, which may facilitate modifications of the host cell wall that permit EVs to enter the host [10]. In addition, fungal EVs are thought to facilitate fungal infection by transporting protein effectors and virulence‐associated molecules to the host cell location [14, 20], which triggers the activation of host immunity and defence responses [6, 21]. They also contain secondary metabolites that may cause damage to host tissues or disrupt plant defences and related signaling mechanisms [17]. For example, Rutter et al. identified proteins linked to the synthesis of secondary metabolites in *C. higginsianum* EVs, including polyketides, nonribosomal peptides, alkaloids, and terpenes, which are frequently implicated in pathogenicity or virulence [17].

However, despite multiple fungal‐plant interaction studies, much remains unknown about exactly how the pathogenic fungal molecules are packaged and transported within EVs to move them across plasma membranes and cell walls for delivery to their functional sites of action. Instead, most studies to date have focused on characterizing the molecular make‐up and downstream functions of the pathogenicity‐associated fungal molecules themselves through fungal transcriptomics [22, 23], proteomics [24, 25], and metabolomics [26] studies. These studies have revealed several key molecules associated with the pathogenicity of fungal species, such as cell wall‐degrading enzymes, secondary metabolites, and effector proteins. Specifically, the expression of several classes of effector molecules [21] has been identified as critical for invasion and disease development in ascomycete fungal pathogens, including necrosis‐ and ethylene‐inducing peptide 1‐like proteins (NLPs) [27], small secreted cysteine‐rich proteins (SSCPs) [28], and apoplastic enzymes [29] that suppress host immunity. There is a growing hypothesis that EVs are responsible for delivering effector and other invasion and defence‐related molecules. For example, *F. graminearum* EVs were found to contain protein effectors (FgHyd3, FGSG_08721, FGSG_03591, FGSG_05292, FGSG_03554, and others), highly expressed during the infection of corn (*Zea mays)*, suggesting that the *F. graminearum* EVs are a mechanism for the unconventional secretion of effectors and virulence factors [14]. Similar evidence was found in *B. cinerea*, where several effector proteins were identified in its EV proteome [12]. Through an in‐depth analysis of the EV structures and contents from specific pathogens, their roles in triggering host recognition and overcoming defence mechanisms are becoming clearer [9, 30]. Furthermore, it may be possible to engineer novel approaches to disease management by understanding the intricate relationship between pathogenic fungal EVs, their contents, and host plant cells [6].

One such disease that would benefit from novel management approaches is Ascochyta blight of chickpea, which is currently managed almost solely by chemical fungicide applications due to an increase in pathogen aggressiveness combined with the erosion of existing host resistance [31]. Chickpea Ascochyta blight is caused by *Ascochyta rabiei* (syn. *Phoma rabiei)*, a haploid, heterothallic fungus belonging to the phylum Ascomycota [32]. The fungal infection induces circular necrotic lesions on leaves and pods that extend to the stems and petioles, resulting in reduced photosynthesis, stem breaking, pod abortion, seed discoloration, and eventual plant death [33]. To date, it is unknown if *A. rabiei* produces EVs and, if it does, if they contain cargo that may be involved in chickpea recognition, signaling for *A. rabiei* invasion, and inciting (or evading) host defence responses. However, this hypothesis is worth investigating since the closely related ascomycete *Z. tritici* was found to secrete EVs containing proteins related to host‐pathogen interaction [15].

Therefore, the aims of this study were to isolate and morphologically characterize *A. rabiei* EVs produced in vitro, as well as to conduct proteomic profiling of these EV protein cargos and predict their functions in the chickpea‐*A. rabiei* pathosystem. This study also examined the variations in the production and cargo of *A. rabiei* EVs with and without the host's presence.

## 2 Materials and methods

### 2.1 Fungal Isolate and Preparation of Inoculum

The *A. rabiei* isolate selected for this study was the highly pathogenic isolate AR0128, which was collected in 2020 from Yarrari, NSW, from the chickpea cultivar PBA Boundary (see Ascochyta dashboard: http://bit.ly/asco‐dashboard). A working culture of the isolate was established by transferring a mycelial agar plug stored in sterile water at 4°C in a refrigerator onto freshly prepared V8 juice agar media plates. The Plates were sealed with parafilm and incubated for 14 days at 20°C ± 2°C with 8 h of darkness and 16 h of light (350–400 nm; Valoya LED grow lights, Finland) at an illumination intensity of approximately 220–250 µmol m^−2^ s^−1^, and inoculum was prepared to a concentration of 10^5^ spores/mL according to the protocol described by Sambasivam et al. (2020) [33].

### 2.2 Plant Materials

Chickpea genotype PBA HatTrick seed was sourced from the Australian National Chickpea Breeding Programme, Tamworth, NSW, Australia. Plants were grown in 15 cm diameter pots using Richgro premium potting mix under a controlled environment maintained at 22°C ± 2°C, with 8 h of darkness and 16 h of light (350–400 nm; Valoya LED grow lights, Finland) at an illumination intensity of approximately 220–250 µmol m^−2^ s^−1^ at Griffith University, Nathan campus, Queensland, Australia. The plants were grown up to maturity and the entire plant foliage was subsequently harvested, air‐dried at 65°C until completely dry, and powdered using an electrical grinder (Target, Australia).

### 2.3 Culture Conditions

The *A. rabiei* isolate was cultured in 100% potato dextrose broth (PDB; Merck, Germany) without and with host (dried chickpea powder added at 8 g/L; PDB+Ch). For this, 250 mL of PDB or PDB+Ch was inoculated with 10^5^ spores/mL of *A. rabiei* AR0128 in a 500 mL flask, which was subsequently incubated at 24°C with 100 rpm agitation for 14 days [7]. All treatments were applied with six biological replicates. Culture media containing only sterile water (instead of the *A. rabiei* inoculum) were used as negative controls.

### 2.4 Optimization of EV‐Like Particle Isolation via Differential Ultracentrifugation and Filtration

**FIGURE 1:**
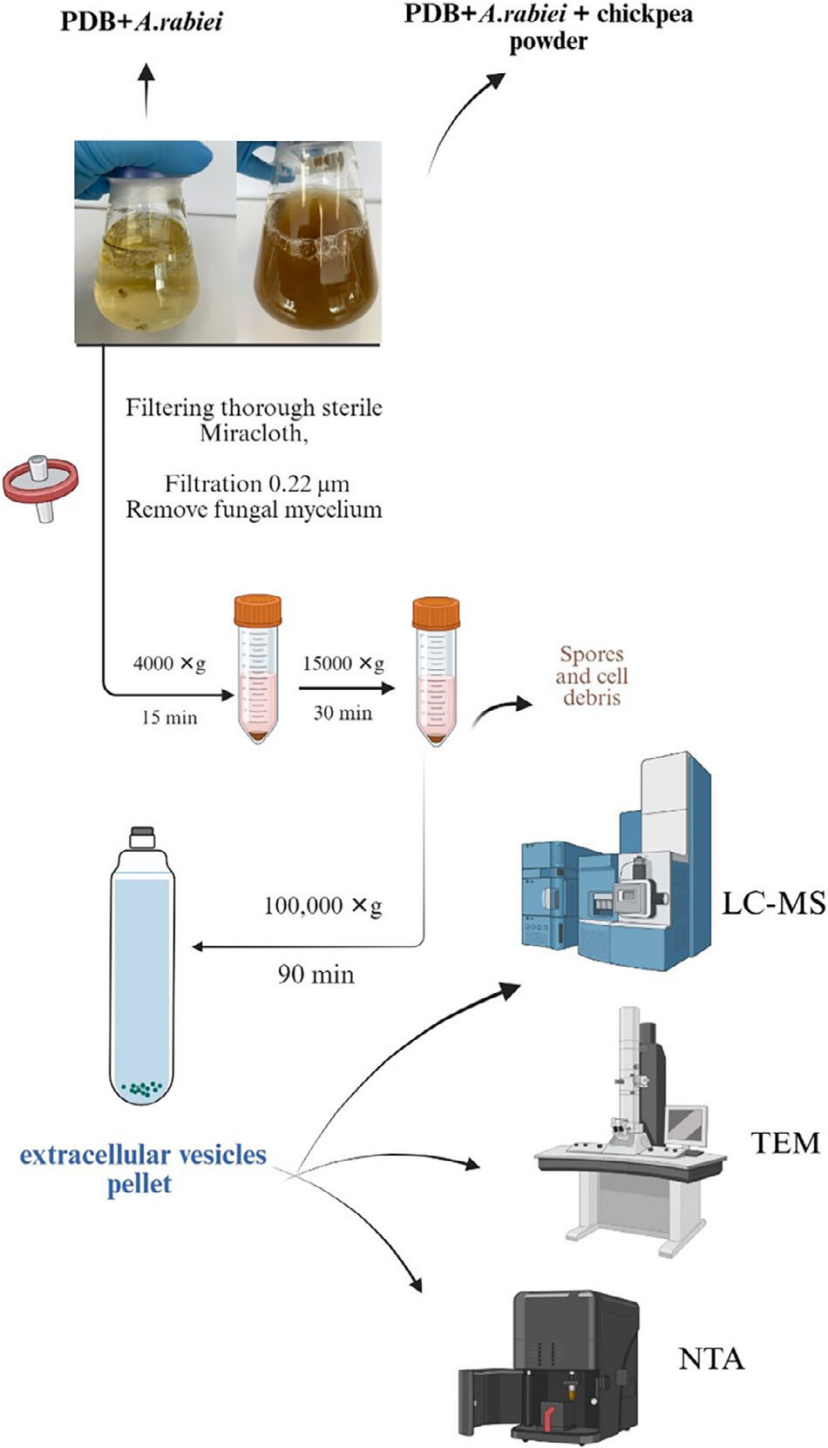
The optimized *A. rabiei* EV isolation protocol. LC‐MS, liquid chromatography–mass spectrometry; NTA, nanoparticle tracking analysis; PDB, potato dextrose broth; TEM, transmission electron microscopy (created with BioRender.com).

To determine if *A. rabiei* produces EVs in broth culture with and without the host, the culture supernatants were filtered and then ultracentrifuged according to protocols developed by Rodrigues et al. (2008) and Bleackley et al. (2020) [7, 34]. Briefly, mycelia were removed from the culture medium with sterile Miracloth and then using a Millex GV syringe filter unit (0.22 µm). After that, spores and cell debris were removed by centrifugation at 4000 × *g* for 15 min, followed by 15,000 × *g* for 30 min through sequentially removing the supernatant from any pelleted mass. The supernatant was further centrifuged at 100,000 × *g* for 90 min at 4°C, using a 70.1 Ti rotor in an Optima L‐90 ultracentrifuge (Beckman Coulter). The final supernatant was discarded using a plastic pipette, without disturbing the pellet (Figure 1), and resultant pellets were resuspended in 50 to 100 µL of sterile phosphate‐buffered saline (PBS; Thermo Fisher Scientific), and samples were stored at 4°C for transmission electron microscopy (TEM) and nanoparticle tracking analysis (NTA) analysis and at −80°C for later molecular analyses.

### 2.5 Transmission Electron Microscopy (TEM)

Samples were prepared following the protocol of Abeysinghe et al. (2023) [35], and observations were made at the Central Analytical Research Facility (CARF) within the Science and Engineering Centre, Queensland University of Technology, Queensland, Australia. Briefly, continuous grids (carbon‐coated, hexagonal mesh size 200, thick grids, ProSciTech) were glow‐discharged using a glow discharge unit (Quorum Technologies) for 60 s. Then, 5 µL of EV samples was transferred to a piece of parafilm. These were then fixed onto carbon 200 mesh copper grids for 3 min, and excess liquid was wicked away with tissue paper. After that, grids were mounted onto a droplet of 2% uranyl acetate (aqueous) for 2 min to create contrast and protect any delicate structures. Finally, excess stain was wicked away, and samples were air‐dried at room temperature. Samples were then visualized with a JEOL 1400 transmission electron microscope (JEOL Japan) operated at 200 kV, and images were captured using a 2 K TVIPS CCD camera (TVIPS, Gauting, Germany). Features of size (30–150 nm) and shape (round‐shaped structures with bilayer membranes and cup‐shaped particles) [7, 18] were used to characterize putative *A. rabiei* EVs.

### 2.6 Nanoparticle Tracking Analysis (NTA)

The sizes and concentrations of putative EVs were subsequently assessed using a NanoSight NS300 instrument equipped with a 405 nm (blue) laser (NanoSight, Ltd., Malvern, UK). For this, samples were diluted in sterile phosphate buffered saline (PBS) and promptly automatically injected into the instrument's loading chamber at an infusion rate of 100. Samples were diluted to achieve 20–200 particles per frame, and image captures were set to occur every 60 s, with three technical replicates for each sample. All analyses were performed at 25°C, and PBS was used as a blank. Data were analyzed using NTA 3.4 Build 3.4.4 software under auto‐analysis settings (NanoSight, Ltd., Malvern, UK).

### 2.7 Sample Preparation for Mass Spectrometry

The total protein concentrations of EV samples (six biological replicates) were initially quantified using the Pierce BCA Protein Assay Kit according to the manufacturer's instructions (Thermo Fisher Scientific Inc., Waltham, MA, USA). Briefly, 2 µL of each sample was combined with 23 µL of 1× PBS and 200 µL of working BCA reagent (supplied in the BCA Protein Assay Kit) in a 96‐well microplate. The resulting mixture was incubated for 30 min at 37°C, and the absorbance was measured at 562 nm using a plate reader (Multiskan skyHigh microplate spectrophotometer, Thermo Fisher Scientific, AU). Protein concentration was calculated against a standard curve prepared with bovine serum albumin (BSA) ranging from 20 to 2000 µg/mL. EV samples containing 12.5 µg of total protein were then prepared for mass spectrometry analysis using the Filter‐Aided Sample Preparation method [36, 37, 38]. Briefly, each EV sample was combined with 15 µL of 4% sodium dodecyl sulphate (SDS)‐Tris buffer (Merck Life Science, Pty Ltd.), 100 mM Tris‐HCl pH 8.5, and 100 mM Dithiothreitol (DTT‐RO Roche, Merck Pty Ltd.). Additionally, 200 µL of DTT‐Urea buffer, containing 25 mM DTT in urea buffer (8 M urea in 100 mM Tris‐HCl pH 8.5), was added. The resultant mixture was then placed in a 30 kDa Microcon YM‐30 centrifugal filter device (Millipore) and incubated at room temperature for 60 min on a Solaris Open Air Orbital Shaker (Thermo Fisher Scientific, AU) at 100 rpm. The buffers were removed by filtering via centrifugal force of 14,000 × *g* at a temperature of 21°C for 15 min. The filters were subsequently rinsed with 200 µL of urea buffer via centrifugation at 14,000 × *g* at 21°C for 15 min. To perform cysteine alkylation, 100 µL of a 50 mM solution of iodoacetamide (IAM) in urea buffer was added to each sample. The samples were then incubated in the dark for 20 min. The filters were washed with 200 µL of urea buffer by centrifugation at 14,000 × *g* for 15 min at 21°C. The buffer was then exchanged by washing the filter twice with 100 µL of 100 mM ammonium bicarbonate. Protein digestion was carried out by adding proteomics‐grade trypsin (Sigma‐Aldrich, St. Louis, MO, USA) at a 1:50 enzyme‐to‐protein ratio and incubating overnight at 37°C. Finally, the digested peptides were collected into sterile 1.5 mL microcentrifuge tubes by centrifugation at 14,000 × *g*. The peptides were then dehydrated using a Speed Vac vacuum concentrator (Thermo Fisher Scientific, AU) and resuspended in 10 µL of 2% acetonitrile (ACN) with 0.1% trifluoroacetic acid (TFA). The resulting samples were cleaned up utilizing STAGE tips with a double SCX membrane (Empore, part no: 2251, 3 M, Maplewood, MI, USA) [39]. Simply put, the STAGE tips were activated by flowing 30 µL of 100% ACN through them and then balanced with 30 µL of 0.2% TFA. The acidified samples were applied onto the STAGE tips and then subjected to three consecutive washes with 30 µL of 0.2% TFA. The samples were extracted with 30 µL of 5% ammonium hydroxide with 80% ACN. Isolated peptides were dehydrated using a SpeedVac Vacuum Concentrator and then mixed with 10 µL of 2% ACN with 0.1% formic acid (FA). The resulting sample was then placed into a polypropylene autosampler vial (Thermo Fisher Scientific, AU). Samples were stored at −20°C for further analysis.

### 2.8 Proteomic Analysis Using Mass Spectrometry (LC‐MS/MS)

Sample processing and analyses were performed to identify EV proteins following the protocol of Zhang et al. (2020) and Müller Bark et al. (2023) [38, 40]. Briefly, the nanoLC‐nanoESI‐MS/MS technique on a TripleTOF 5600+ instrument (SCIEX) was used to obtain peptide spectrum data from roughly 400 ng–1 µg of injected tryptic peptides per sample. The peptides were separated using reversed‐phase chromatography with an Eksigent ekspert nanoLC 400 System, which was immediately connected to the MS/MS equipment. The LC platform was arranged in a trap and elute configuration, consisting of a 10 mm × 0.3 mm trap cartridge filled with ChromXP C18CL 5 µm 120 Å material and a 150 mm × 75 µm analytical column packed with ChromXP C18 3 µm 120 Å material (Eksigent Technologies, Dublin, CA). The mobile phase solvents comprised three components: Mobile Phase A, which was a mixture of water and 0.1% FA; Mobile Phase B, which was a mixture of ACN and 0.1% FA; and Mobile Phase C, which was a mixture of water, 2% ACN, and 0.1% FA. Trapping was conducted in Mobile Phase C for a duration of 5 min at a flow rate of 5 uL/min. The separation process utilized two mobile phases, A and B, which flowed at a constant rate of 300 nL/min. The proportions of solvents A and B were adjusted at specific time points (0, 30, 35, 40, 49, 50, and 60 min) according to the following percentages: 98% A, 60% A, 35% A, 10% A, 10% B, 98% B, and 98% A. To reduce the occurrence of changes in retention time, the analytical column was kept at a constant temperature of 40°C. To account for variation in peptide load (400 ng–1 µg), protein quantification and normalization were performed using the global bias correction algorithm implemented in ProteinPilot (SCIEX). This ensured that protein abundances were comparable across samples and treatments.

### 2.9 Bioinformatic Analysis of Proteomic Dataset

After chromatography, peptides were analyzed by data‐dependent acquisition (DDA). The TripleTOF 5600+ instrument (SCIEX) was configured in DDA mode to acquire high‐resolution (30,000) TOF‐MS scans spanning a range of 350–1350 m/z. This was followed by 40 MS/MS scans with high sensitivity, targeting the most prevalent peptide ions in each cycle within the range of 100–2000 m/z [40]. The peptide ions were selected based on two criteria: (1) an intensity greater than 150 cps, and (2) a charge state between 2 and 5. The time for dynamic exclusion was set to 9 s. The subsequent product ion (MS/MS) scan was acquired for 50 ms, and each survey (TOF‐MS) scan lasted 250 ms, for a total cycle duration of 2.3 s. Rolling collision energy was used in the collision cell to break up the ions, and the collision energy spread (CES) was adjusted to 5. The peptide ion fragmentation spectra that were gathered were saved in SCIEX files in wiff.scan format [38]. Then, peptide identification was performed by the Protein Pilot 5.0 software version 5.0.2 (AB SCIEX) using the Uni‐Prot/Swiss‐Prot database with the following setting: Sample Type: identification; cysteine alkylation: iodoacetamide; instrument: TripleTof 5600; Species: None; ID focus: Biological modification; Search effort: thorough ID; Detected protein threshold: 0.05.

The *A. rabiei* protein database was downloaded from https://www.uniprot.org/proteomes/UP000076837 on 10th December 2024) for *A. rabiei* protein identification [41]. The generated protein list was filtered manually by excluding proteins with a single matching peptide, proteins found in only one biological replicate, or proteins detected in media without *A. rabiei* (negative controls). For SWATH‐MS data acquisition and label‐free quantitation (LFQ), the LC settings were similar to those utilized for DDA. The mass spectrometry acquisition protocol was as outlined in Gillet et al. (2012) [42]. A protein library was created using the DDA data as outlined above for focused data extraction and peptide quantification. The abundance of peptides was measured using PeakView Software version 2.2 (AB SCIEX), with standard settings as described in Zacchi and Schulz (2016) [43]. Matches with *p* ≤ 0.05 were considered significant.

### 2.10 Gene Ontology (GO) Analysis and Prediction of Effector Proteins in EV‐Like Particle Samples

The reference genome of *A. rabiei* in Uniprot (UP000076837, assembly GCA_001630375.1) was loaded into OmicsBox to annotate proteins with GO terms, using the default settings [44]. The resulting list was used as a reference to perform GO analyses on proteins isolated from different culture media, using Fisher's exact test. The “reduce to most specific” function was applied to all analyses. GO terms with a *p* value below 0.05 were considered overrepresented. The identified proteins were also submitted to ApoplastP [45] for apoplastic localization, to SignalP‐6.0 [46] for signal peptide prediction, to Deep Loc‐2.1 [47] for subcellular localization prediction, and EffectorP‐3.0 for effector protein prediction [48].

### 2.11 Functional Comparison of *A. rabiei* EVs With Other Fungal Pathogens

Published fungal EV proteomes were collated to enable comparative analysis with *A. rabiei*. Two groups of fungi were included:

Group one: Plant‐pathogenic fungi (*F. oxysporum*, *B. cinerea*, *Z. tritici*), which were selected because their EV proteome datasets are available and they represent agronomically important pathogens with infection strategies (necrotrophic/hemibiotrophic) that overlap with *A. rabiei*, and thus provide a biologically relevant context for comparison [10, 12, 15].

Group two: Human‐pathogenic fungi (*Candida albicans, Cryptococcus neoformans, Aspergillus fumigatus*), which were included because their EV proteomes are the most extensively characterized in the literature and provide a reference for conserved EV‐associated proteins across pathogenic fungi [34, 49, 50, 51].

Proteins reported in the selected studies were annotated according to functional descriptions provided in the original publications and verified against UniProt annotations where necessary. For cross‐dataset comparison, proteins were grouped into ten higher‐order functional categories: (1) translation & ribosome, (2) ATP/GTP binding and helicases, (3) protein folding and chaperones, (4) energy and mitochondria, (5) cell wall remodeling (β‐glucan metabolism, glycosyltransferases), (6) RNA processing and spliceosome/snoRNP, (7) vesicle trafficking and membrane microdomains, (8) signaling including calcineurin, and calmodulin‐dependent kinases, (9) cytoskeleton, and (10) carbon and amino‐acid metabolism.

For *A. rabiei*, functional information was derived from the GO enrichment analyses performed for EVs isolated from PDB (host‐absent) and PDB supplemented with chickpea tissue (host‐present). Enriched GO terms were assigned to the same ten categories. Each category was scored for presence or absence across the selected species, and for condition‐specific enrichment in *A. rabiei*. Comparative matrices and the categorical heatmap were generated using Python Software (Version 3.11; Python Software Foundation, 2023) employing the pandas’ library for data handling and matplotlib for visualization.

## 3 Results

### 3.1 Impact of Host and Culture Media Factors on *A. rabiei* EV‐Like Particle Production

**FIGURE 2:**
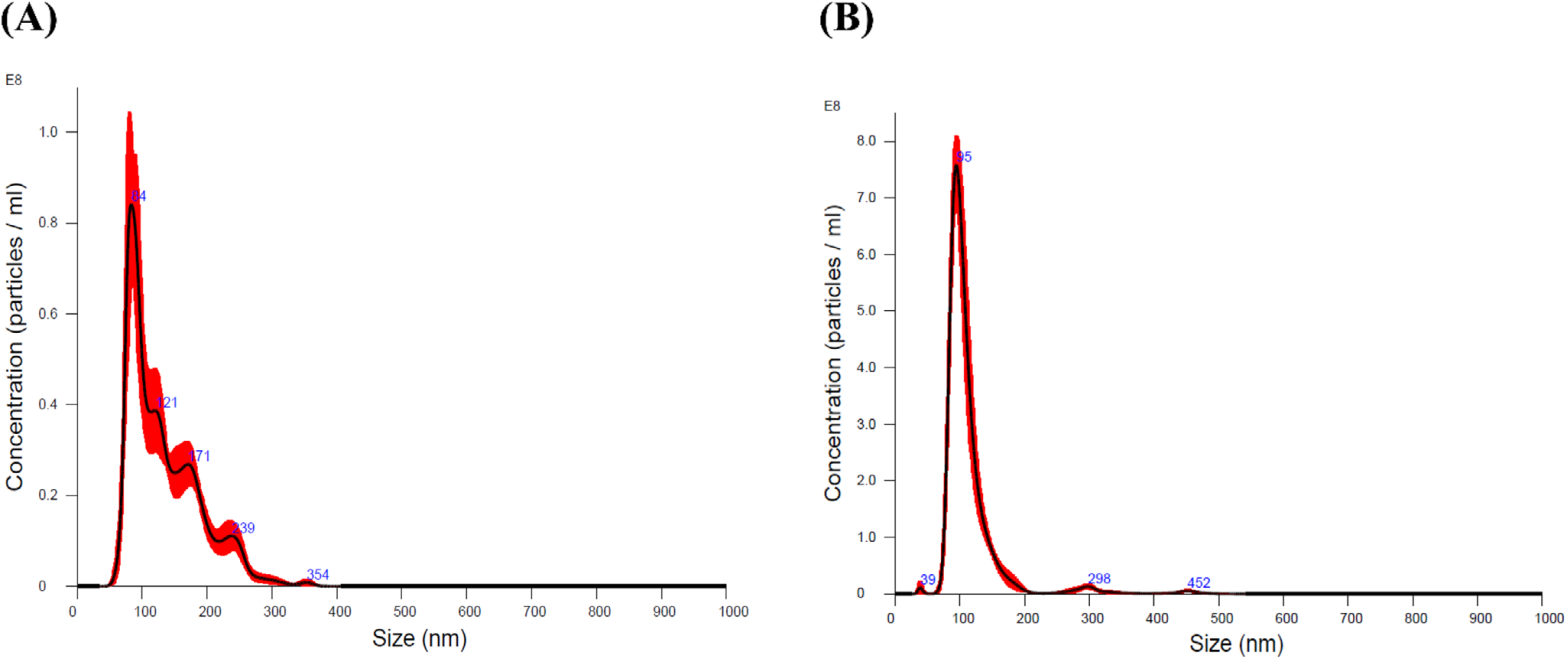
Nanoparticle tracking analysis results of putative EVs purified from the culture supernatant of *A. rabiei*. (A) shows the number and size distribution of the isolated EVs from PDB culture media without chickpea powder, and (B) shows the number and size distribution of the isolated EVs from PDB culture media with chickpea powder. Data are representative of three independent replicates.

EVs were obtained from culture supernatants of *A. rabiei* grown in PDB, produced 5.85 × 10^9^ ± 1.68 × 10^8^ particles/mL of culture with a mean size of 133.7 nm ± 3.4 nm and a mode size of 84.6 nm ± 2.3 nm (Figure 2A). When *A. rabiei* was grown in culture media containing powdered chickpea, 3.01 × 10^10^ ± 3.83 × 10^9^ particles/mL with a mean size of 112.5 nm ±1.5 nm and a mode size of 94.6 nm ± 0.8 nm were produced (Figure 2B). Meaning that there were significantly more EV‐like particles produced by *A. rabiei* when grown with chickpea powder (PDB+Ch), but these were smaller than when grown without chickpea.

**FIGURE 3:**
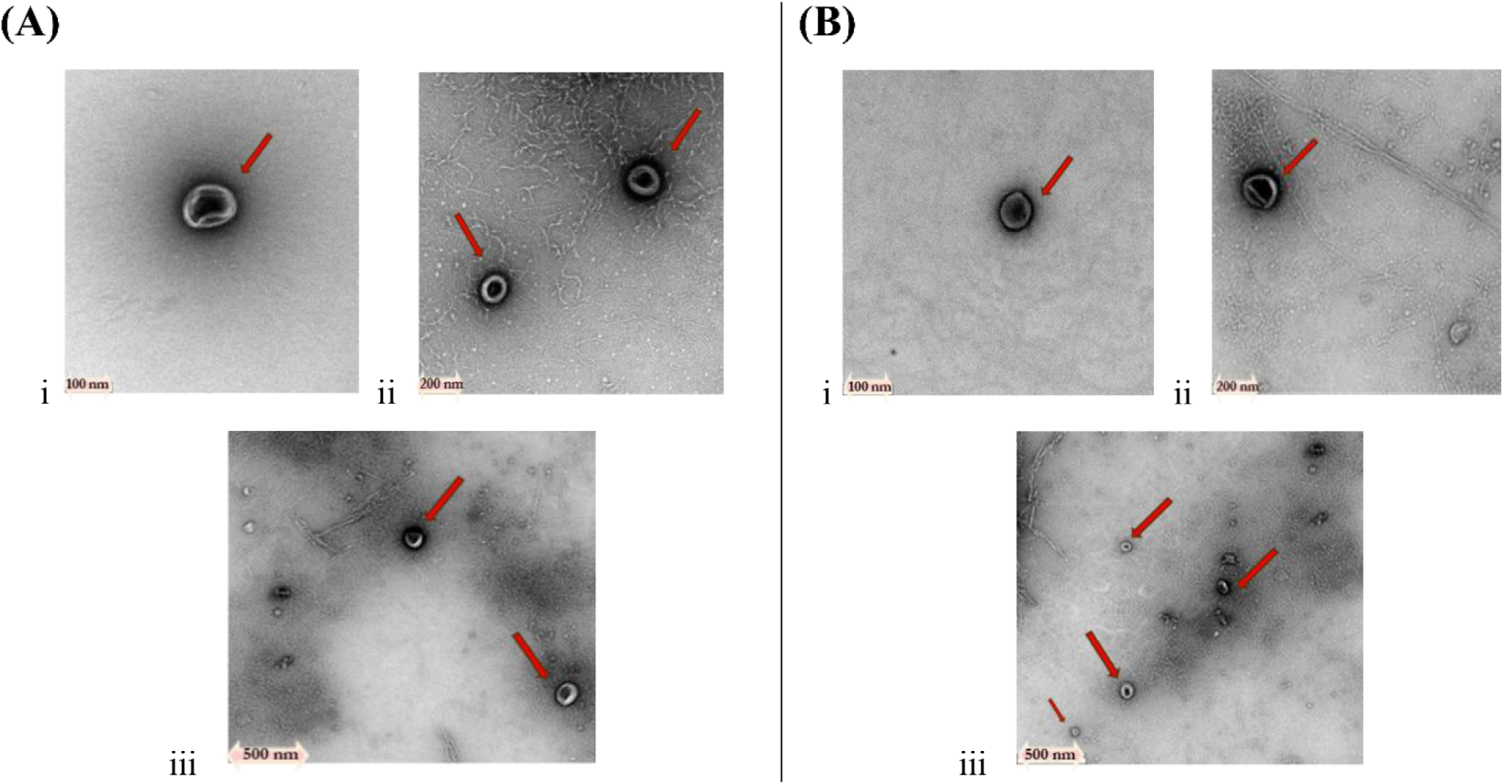
Transmission electron microscopy characterization of particles produced by *A. rabiei*. (A) demonstrates the cup‐like morphology of those isolated from PDB culture media without chickpea powder, and (B) demonstrates the cup‐like morphology of those isolated from PDB culture media with chickpea powder. Images are representative of three independent replicates for each culture media (i–iii).

Meanwhile, TEM analyses demonstrated that the isolated particles displayed the expected characteristics of EVs with sphere and cup‐shaped morphology and a heterogeneous size ranging from approximately 40 to 200 nm for those produced with and without the presence of the host (Figure 3A,B, respectively). Together, the data indicated that the particles isolated from *A. rabiei* possessed common morphological characteristics associated with EVs from other pathogenic fungal species.

### 3.2 The Protein Cargo of *A. rabiei* EV‐Like Particles

The total protein concentrations isolated from *A. rabiei* particles grown in PDB or PDB+Ch, were 0.634 ± 0.073 µg/µL or 1.559 ± 0.242 µg/µL (n = 6), respectively. From these, 276 *A. rabiei* associated proteins were derived from *A. rabiei* particles following growth without the host (PDB; Table S1), and 517 *A. rabiei* associated proteins were derived from *A. rabiei* particles following growth with the host (PDB+Ch; Table S2). Of these, 234 proteins were shared among the treatment datasets.

#### 3.2.1 *A. rabiei* EV‐Like Particle Derived Proteins Found in Media Without the Host (PDB)

**FIGURE 4:**
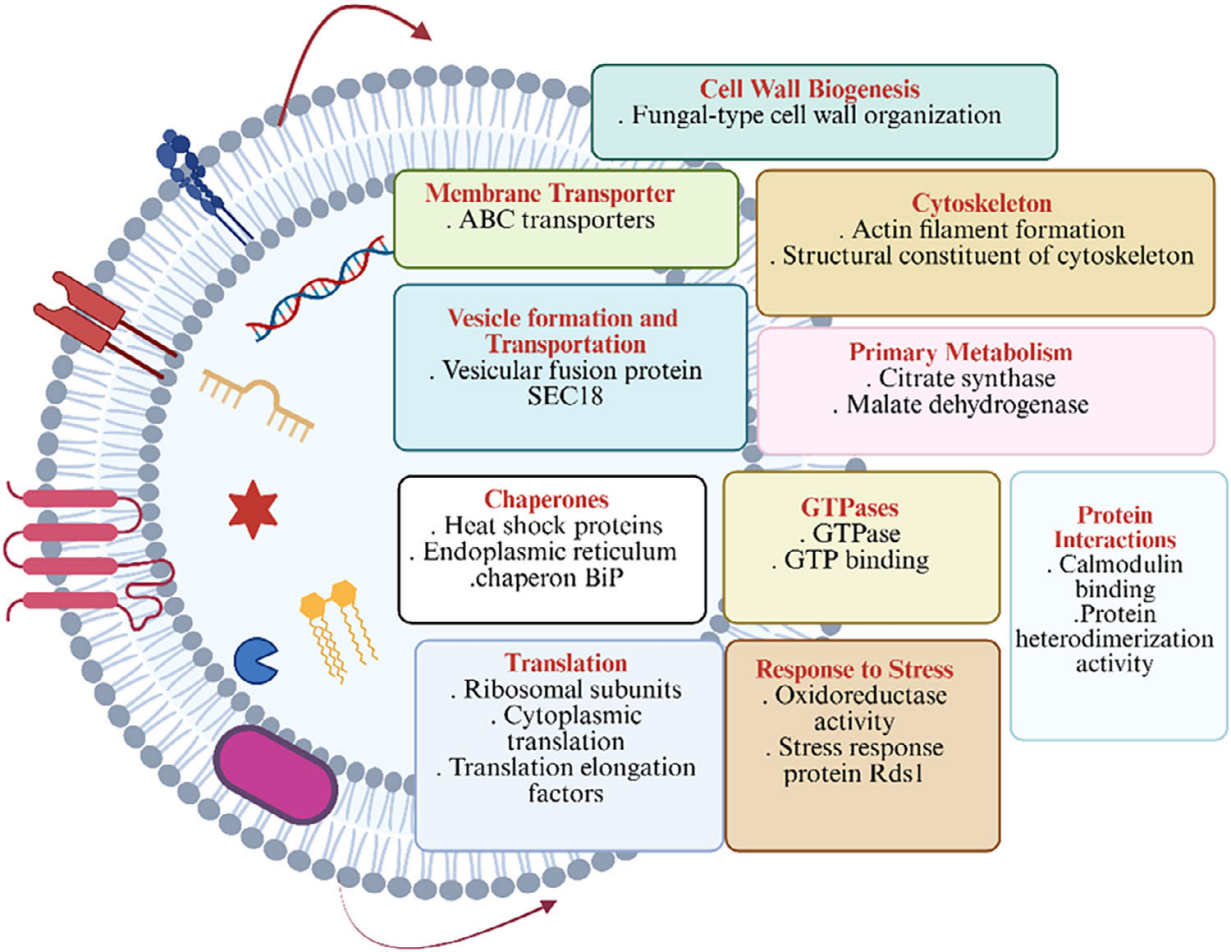
Overview of categories of EV proteins found in *A. rabiei* cultured in PDB.

All GO comparisons were performed against the complete *A. rabiei* proteome downloaded from the Uniprot database. Functional enrichment analysis of the EV proteins in the absence of the host (PDB), identified 87 GO terms that were enriched with an adjusted *p* value cutoff of 0.05 (Table S3). The enriched GO terms give a broader picture of the annotated functions, cellular locations, and biological processes that were overrepresented in the *A. rabiei* EVs. An overview of categories of EV proteins found in PDB is shown in Figure 4.

**FIGURE 5:**
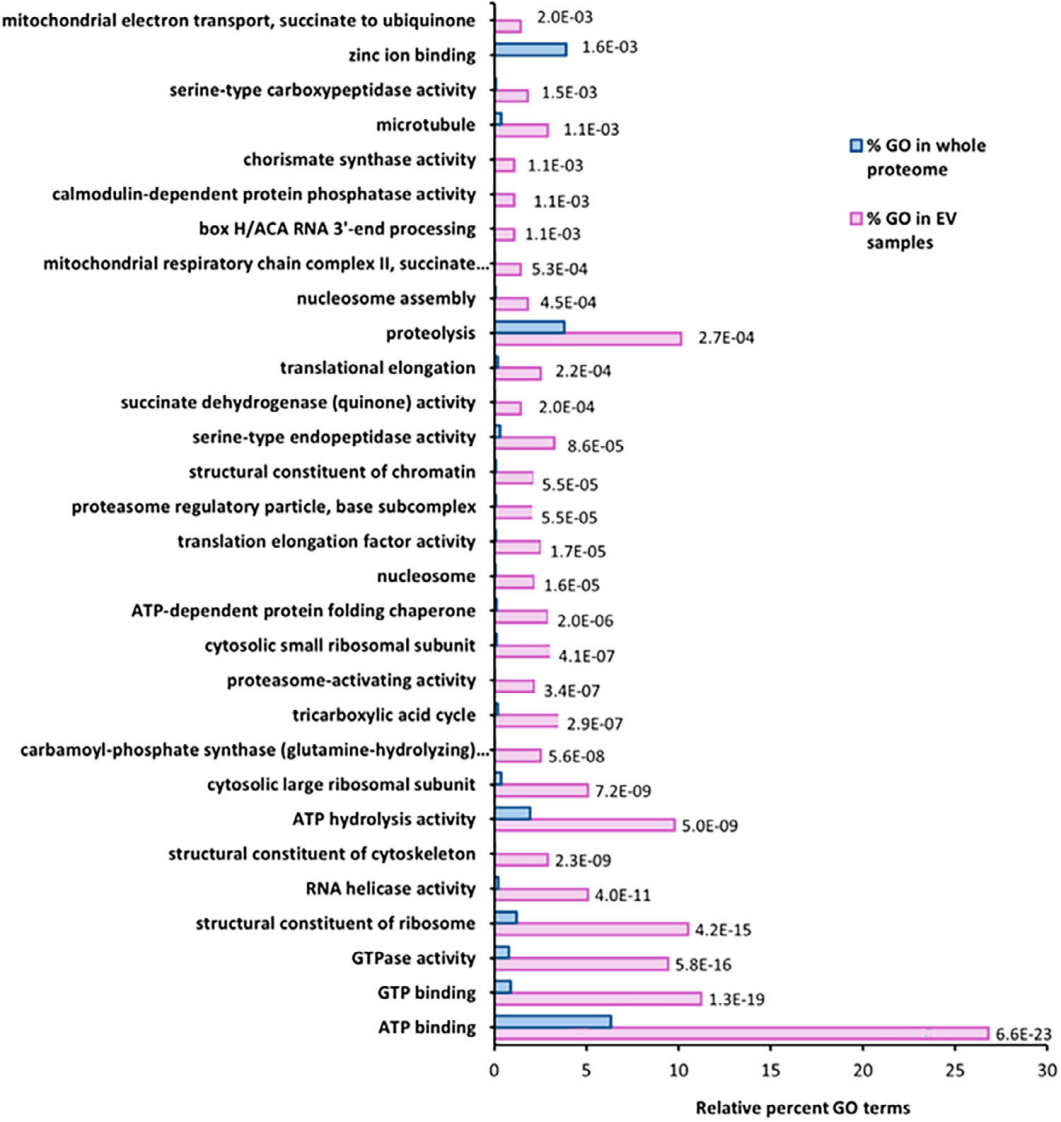
Top 30 gene ontology terms over‐ and under‐represented in *A. rabiei* EV proteins compared to the whole proteome cultured in PDB; full lists can be found in Table S3. The number at the end of the bars is the *p* value for each gene ontology comparison with the proteome.

The overrepresented GO terms in EV proteins in PDB were ATP & GTP‐related proteins, ribosomal proteins, RNA helicase, and other activities (Figure 5; Table S3). Meanwhile, underrepresented GO terms compared to the *A. rabiei* proteome included those related to DNA‐binding transcription factor activity, membrane components, zinc ion binding, and regulation of DNA‐templated transcription (Figure 5; Table S3).

The most abundantly detected proteins in particles recovered from PDB grown *A. rabiei* included cell wall degrading enzymes that facilitate the entry of fungi inside the host cells (endo‐1,4‐beta‐xylanase, cellulase, glucan endo‐1,3‐beta‐glucosidase eglC), enzymes involved in stress adaptation (endoplasmic reticulum chaperone BiP, ATP synthase subunit alpha and beta, HTH TetR‐type domain‐containing protein, glutamate dehydrogenase, GTP‐Binding nuclear protein gsp1/Ran, ADP/ATP Translocase, ATP‐dependent protein folding chaperone), proteins involved in fungal virulence (glutamate dehydrogenase, transaldolase, tripeptidyl‐peptidase, HTH TetR‐Type domain‐containing protein), protein folding (endoplasmic reticulum chaperone BiP, ATP‐dependent protein folding chaperone), protein involved in transport (GTP‐binding nuclear protein gsp1/Ran, ADP/ATP Translocase), protein involved in degradation (tripeptidyl‐peptidase I, serine‐type carboxypeptidase), proteins involved in energy metabolism and ATP processing, and finally, ribosome and translation‐related proteins. A proteolytic enzyme, which plays multiple roles in protein degradation, fungal development, virulence, and host‐pathogen interactions, was also identified. An additional six proteins were not characterized (Table 1).

**TABLE 1:** The most abundantly detected proteins in particles recovered from PDB grown *A. rabiei*.

UniprotKB	UniProc	Protein name	Associated GO terms
A0A976NBJ3	UPI00220DD514	Endo‐1,4‐beta‐xylanase	polysaccharide catabolic process [GO:0000272]; endo‐1,4‐beta‐xylanase activity [GO:0031176]; polysaccharide catabolic process [GO:0000272]; endo‐1,4‐beta‐xylanase activity [GO:0031176]
A0A162VSR6	UPI0007BBEE35	ATP synthase subunit alpha	proton‐transporting ATP synthase complex, catalytic core F(1) [GO:0045261]; ATP binding [GO:0005524]; proton‐transporting ATP synthase activity, rotational mechanism [GO:0046933]
A0A163M692	UPI0007BB79E4	Adenosyl homocysteinase	hydrolase activity [GO:0016787]; one‐carbon metabolic process [GO:0006730]
A0A162ZVX0	UPI0007BBCCDE	ATP synthase subunit beta	proton‐transporting ATP synthase complex, catalytic core F(1) [GO:0045261]; ATP binding [GO:0005524]; proton‐transporting ATP synthase activity, rotational mechanism [GO:0046933]; proton‐transporting ATPase activity, rotational mechanism [GO:0046961]
A0A976NBQ1	UPI00220C4037	Uncharacterized protein	
A0A163G6K6	UPI0007BBAF3D	Cellulase	cellulase activity [GO:0008810]; polysaccharide catabolic process [GO:0000272]
A0A163L1D0	UPI0007BAE431	Endoplasmic reticulum chaperone BiP	endoplasmic reticulum [GO:0005783]; ATP binding [GO:0005524]; ATP‐dependent protein folding chaperone [GO:0140662]
A0A162XWT1	UPI0007BC7CCD	rRNA binding	small ribosomal subunit [GO:0015935]; rRNA binding [GO:0019843]; structural constituent of ribosome [GO:0003735]; translation [GO:0006412]
A0A8H7JWD8	UPI00190246A2	Uncharacterized protein	
A0A8H7K0U8	UPI001902899A	Translation elongation factor 2	GTP binding [GO:0005525]; GTPase activity [GO:0003924]; translation elongation factor activity [GO:0003746]
A0A097PUH1	UPI0000DD353C	ATP binding	cytoskeleton [GO:0005856] hydrolase activity [GO:0016787]
A0A976RGH5	UPI002205CF66	60S ribosomal protein L11	ribonucleoprotein complex [GO:1990904]; ribosome [GO:0005840]; structural constituent of ribosome [GO:0003735]; translation [GO:0006412]
A0A163CNT5	UPI0007BB7EA5	40S ribosomal protein S26	ribonucleoprotein complex [GO:1990904]; ribosome [GO:0005840]; structural constituent of ribosome [GO:0003735]; translation [GO:0006412]
A0A162VT75	UPI0007BB357A	Uncharacterized protein	
A0A163IX09	UPI0007BC5735	ADP/ATP translocase	mitochondrial inner membrane [GO:0005743]; ATP:ADP antiporter activity [GO:0005471]; mitochondrial ADP transmembrane transport [GO:0140021]; mitochondrial ATP transmembrane transport [GO:1990544]
A0A163CE82	UPI0007BC536E	Transaldolase	cytoplasm [GO:0005737]; transaldolase activity [GO:0004801]; carbohydrate metabolic process [GO:0005975]; pentose‐phosphate shunt [GO:0006098]
A0A163J7I9	UPI00000013B2	Histone H4	nucleosome [GO:0000786]; nucleus [GO:0005634]; DNA binding [GO:0003677]; protein heterodimerization activity [GO:0046982]; structural constituent of chromatin [GO:0030527]
A0A976NAE7	UPI00220CE9E8	GTP‐binding nuclear protein gsp1/Ran	nucleus [GO:0005634]; GTP binding [GO:0005525]; GTPase activity [GO:0003924]; nucleocytoplasmic transport [GO:0006913]; protein transport [GO:0015031]
A0A163L5Q8	UPI0004A92A0D	HTH tetR‐type domain‐containing protein	DNA binding [GO:0003677]
A0A8H7JLU1	UPI001901FBFA	Uncharacterized protein	
A0A8H7JUP4	UPI0018FF6300	Tripeptidyl‐peptidase I	extracellular space [GO:0005615]; metal ion binding [GO:0046872]; serine‐type endopeptidase activity [GO:0004252]; proteolysis [GO:0006508]
A0A163FBF3	UPI0007BB5879	Serine‐type carboxypeptidase	serine‐type carboxypeptidase activity [GO:0004185]; proteolysis [GO:0006508]
A0A162VCT1	UPI0007BB2ADE	Structural constituent of ribosome	cytosolic large ribosomal subunit [GO:0022625]; RNA binding [GO:0003723]; structural constituent of ribosome [GO:0003735]
A0A162YZK1	UPI0007BC34D3	RNA binding	small ribosomal subunit [GO:0015935]; RNA binding [GO:0003723]; structural constituent of ribosome [GO:0003735]; translation [GO:0006412]
A0A163H285	UPI0007BB81ED	Glutamate dehydrogenase	glutamate dehydrogenase (NADP+) activity [GO:0004354]; nucleotide binding [GO:0000166]; amino acid metabolic process [GO:0006520]
A0A163DU27	UPI0007BB3E2C	Histone H2B	nucleosome [GO:0000786]; nucleus [GO:0005634]; DNA binding [GO:0003677]; protein heterodimerization activity [GO:0046982]; structural constituent of chromatin [GO:0030527]
A0A162Y8Z3	UPI0007BB2637	40S ribosomal protein S4	ribonucleoprotein complex [GO:1990904]; ribosome [GO:0005840]; rRNA binding [GO:0019843]; structural constituent of ribosome [GO:0003735]; translation [GO:0006412]
A0A976NJF2	UPI0021FA6A6F	Uncharacterized protein	
A0A162×5C7	UPI0007BB11A7	ATP binding	ATP binding [GO:0005524]; ATP‐dependent protein folding chaperone [GO:0140662]; protein refolding [GO:0042026]
A0A8H7MV62	UPI001900610C	Uncharacterized protein	

#### 3.2.2 *A. rabiei* EV‐Like Particle Derived Proteins Found in Media With the Host (PDB+Ch)

**FIGURE 6:**
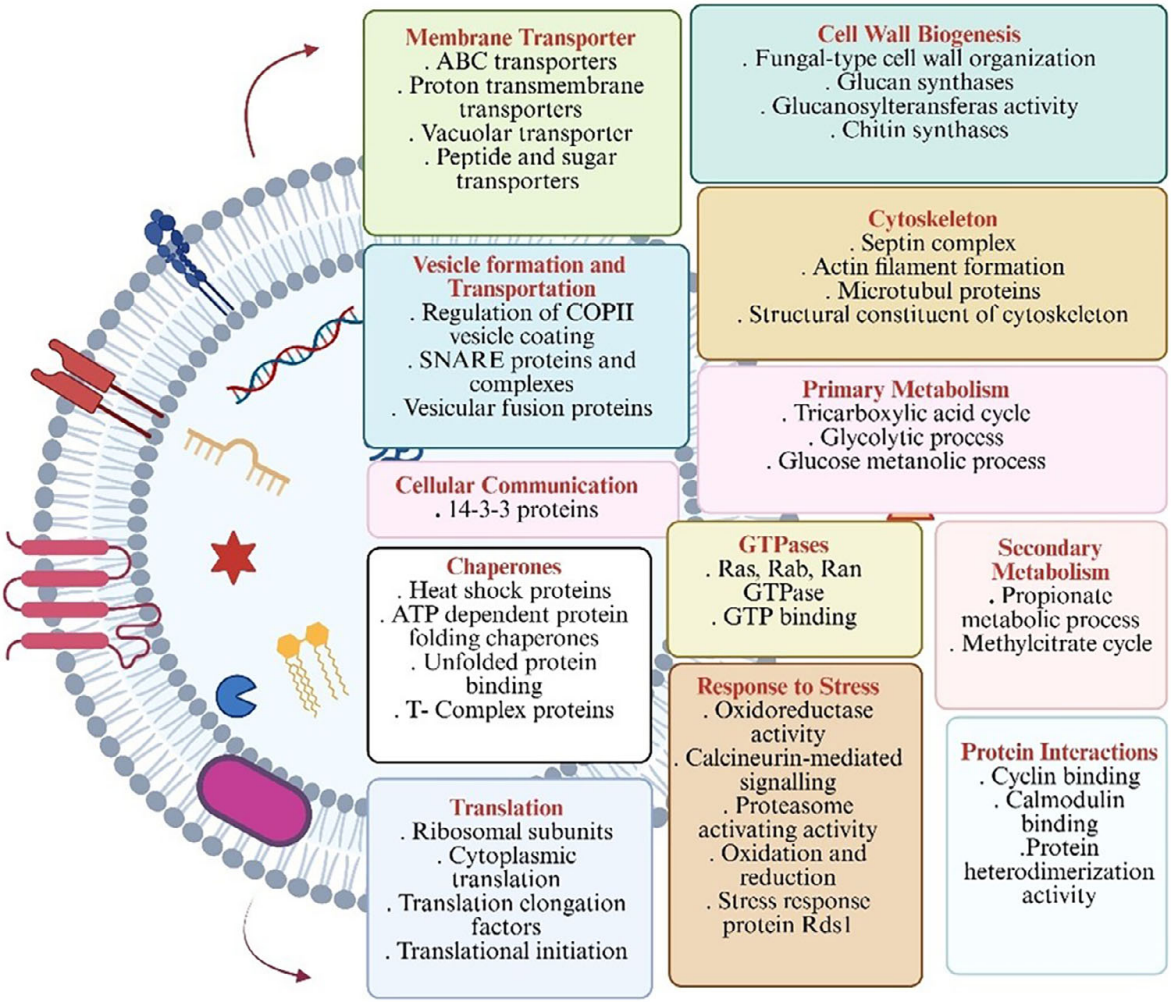
Overview of categories of EV proteins found in *A. rabiei* cultured in PDB with chickpea powder.

Functional enrichment analysis of the EV proteins in PDB+Ch identified 135 GO terms that were enriched with an adjusted *p* value cutoff of 0.05 (Table S4). An overview of categories of EV proteins found in PDB+Ch is shown in Figure 6.

**FIGURE 7:**
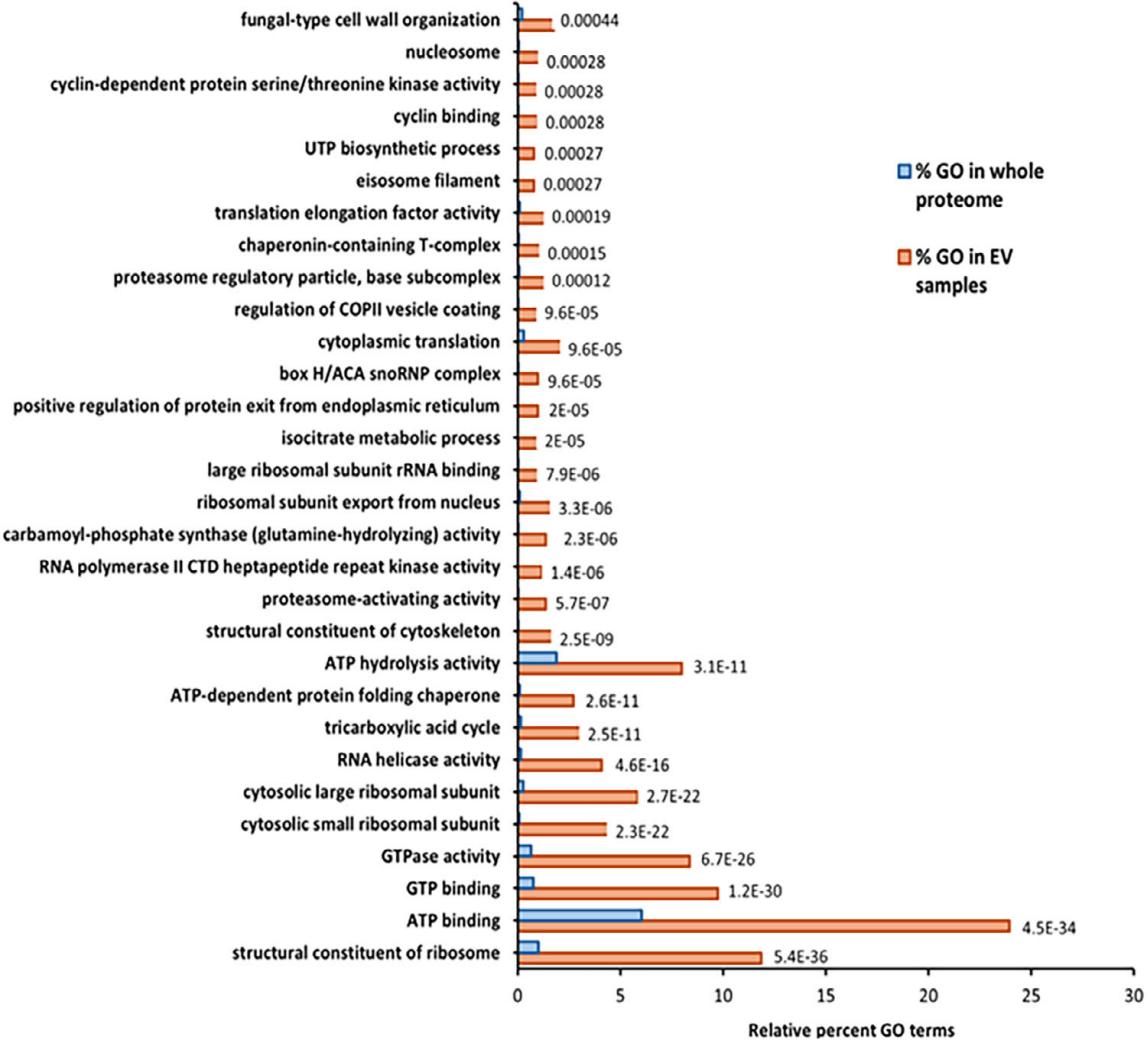
Top 30 gene ontology terms over‐ and under‐represented in *A. rabiei* EV proteins compared to the whole proteome in PDB with chickpea powder; full lists can be found in Table S4. The number at the end of the bars is the *p* value for each gene ontology comparison with the proteome.

Similar to the samples grown without the host (PDB), these were associated with ATP & GTP‐related, ribosomal, RNA helicase, and other activities, including the tricarboxylic acid cycle. Meanwhile, underrepresented proteins included those involved in heme binding, zinc ion binding, and monooxygenase activity (Figure 7; Table S4).

The most abundantly detected proteins from particles grown with the host (PDB+Ch) included the cell wall degrading enzymes (endo‐1,4‐beta‐xylanase), enzymes involved in stress adaptation (endoplasmic reticulum chaperone BiP, ATP synthase subunit alpha and beta, ADP/ATP translocase, glutamate dehydrogenase, adenosylhomocysteinase, transaldolase, tripeptidyl‐peptidase I, ATP binding protein, GTP‐binding nuclear protein gsp1/Ran), fungal virulence proteins (glutamate dehydrogenase, transaldolase, tripeptidyl‐peptidase), proteins involved in protein folding (endoplasmic reticulum chaperone BiP, ATP binding protein), transport, and degradation (endoplasmic reticulum chaperone BiP, Tripeptidyl‐peptidase), proteins involved in energy metabolism and ATP processing and also ribosome and translation‐related proteins. A further eight proteins were uncharacterized (Table 2).

**TABLE 2:** The most abundantly detected proteins in particles recovered from PDB+Ch grown *A. rabiei*.

UniprotKB	UniProc	Protein name	Associated GO terms
A0A976NBJ3	UPI00220DD514	Endo‐1,4‐beta‐xylanase	endo‐1,4‐beta‐xylanase activity [GO:0031176]; polysaccharide catabolic process [GO:0000272]
A0A163IX09	UPI0007BC5735	ADP/ATP translocase	mitochondrial inner membrane [GO:0005743]; ATP:ADP antiporter activity [GO:0005471]; mitochondrial ADP transmembrane transport [GO:0140021]; mitochondrial ATP transmembrane transport [GO:1990544]
A0A162VSR6	UPI0007BBEE35	ATP synthase subunit alpha	proton‐transporting ATP synthase complex, catalytic core F(1) [GO:0045261]; ATP binding [GO:0005524]; proton‐transporting ATP synthase activity, rotational mechanism [GO:0046933]
A0A8H7JUP4	UPI0018FF6300	Tripeptidyl‐peptidase I	extracellular space [GO:0005615]; metal ion binding [GO:0046872]; serine‐type endopeptidase activity [GO:0004252]; proteolysis [GO:0006508]
A0A162ZVX0	UPI0007BBCCDE	ATP synthase subunit beta	proton‐transporting ATP synthase complex, catalytic core F(1) [GO:0045261]; ATP binding [GO:0005524]; proton‐transporting ATP synthase activity, rotational mechanism [GO:0046933]; proton‐transporting ATPase activity, rotational mechanism [GO:0046961]
A0A162VT75	UPI0007BB357A	Uncharacterized protein	
A0A163JDZ1	UPI0007BB8383	ATP binding	cytoplasm [GO:0005737]; proteasome regulatory particle, base subcomplex [GO:0008540]; ATP binding [GO:0005524]; ATP hydrolysis activity [GO:0016887]; proteasome‐activating activity [GO:0036402]; protein catabolic process [GO:0030163]
A0A976NBQ1	UPI00220C4037	Uncharacterized protein	
A0A163M692	UPI0007BB79E4	Adenosylhomocysteinase	hydrolase activity [GO:0016787]; one‐carbon metabolic process [GO:0006730]
A0A976RGH5	UPI002205CF66	60S ribosomal protein L11	ribonucleoprotein complex [GO:1990904]; ribosome [GO:0005840]; structural constituent of ribosome [GO:0003735]; translation [GO:0006412]
A0A163L1D0	UPI0007BAE431	Endoplasmic reticulum chaperone BiP	endoplasmic reticulum [GO:0005783]; ATP binding [GO:0005524]; ATP‐dependent protein folding chaperone [GO:0140662]
A0A8H7JWD8	UPI00190246A2	Uncharacterized protein	
A0A162XWT1	UPI0007BC7CCD	rRNA binding	small ribosomal subunit [GO:0015935]; rRNA binding [GO:0019843]; structural constituent of ribosome [GO:0003735]; translation [GO:0006412]
A0A163CE82	UPI0007BC536E	Transaldolase	cytoplasm [GO:0005737]; transaldolase activity [GO:0004801]; carbohydrate metabolic process [GO:0005975]; pentose‐phosphate shunt [GO:0006098]
A0A8H7K0U8	UPI001902899A	Translation elongation factor 2	GTP binding [GO:0005525]; GTPase activity [GO:0003924]; translation elongation factor activity [GO:0003746]
A0A097PUH1	UPI0000DD353C	ATP binding	cytoskeleton [GO:0005856]; hydrolase activity [GO:0016787]
A0A8H7MV62	UPI001900610C	Uncharacterized protein	
A0A163CNT5	UPI0007BB7EA5	40S ribosomal protein S26	ribonucleoprotein complex [GO:1990904]; ribosome [GO:0005840]; structural constituent of ribosome [GO:0003735]; translation [GO:0006412]
A0A162WCN9	UPI0007BBE00E	Uncharacterized protein	
A0A163BJM6	UPI0007BB475E	Structural constituent of ribosome	RNA binding [GO:0003723]; structural constituent of ribosome [GO:0003735]
A0A162YZK1	UPI0007BC34D3	RNA binding	small ribosomal subunit [GO:0015935]; RNA binding [GO:0003723]; structural constituent of ribosome [GO:0003735]; translation [GO:0006412]
A0A8H7JLU1 (Homolog: A0A163MLY3)	UPI001901FBFA (Homolog: UPI0007BB33D0)	Uncharacterized protein (Homolog: Probable glucan endo‐1,3‐beta‐glucosidase eglC)	cell surface [GO:0009986]; extracellular region [GO:0005576]; fungal‐type cell wall [GO:0009277]; plasma membrane [GO:0005886]; side of membrane [GO:0098552]; glucan endo‐1,3‐beta‐D‐glucosidase activity [GO:0042973]; cell wall organization [GO:0071555]; polysaccharide catabolic process [GO:0000272]
A0A976NAE7	UPI00220CE9E8	GTP‐binding nuclear protein gsp1/Ran	nucleus [GO:0005634]; GTP binding [GO:0005525]; GTPase activity [GO:0003924]; nucleocytoplasmic transport [GO:0006913]; protein transport [GO:0015031]
A0A163MK75	UPI0007BB3A9F	Uncharacterized protein	
A0A163J7I9	UPI00000013B2	Histone H4	nucleosome [GO:0000786]; nucleus [GO:0005634]; DNA binding [GO:0003677]; protein heterodimerization activity [GO:0046982]; structural constituent of chromatin [GO:0030527]
A0A976NHA5	UPI001A24083C	Ribosomal 40S subunit protein S18B	ribonucleoprotein complex [GO:1990904]; ribosome [GO:0005840]; RNA binding [GO:0003723]; structural constituent of ribosome [GO:0003735]; translation [GO:0006412]
A0A163H285	UPI0007BB81ED	Glutamate dehydrogenase	glutamate dehydrogenase (NADP+) activity [GO:0004354]; nucleotide binding [GO:0000166]; amino acid metabolic process [GO:0006520]
A0A163MIG1	UPI0007BC04BB	Uncharacterized protein	
A0A163DU27	UPI0007BB3E2C	Histone H2B	nucleosome [GO:0000786]; nucleus [GO:0005634]; DNA binding [GO:0003677]; protein heterodimerization activity [GO:0046982]; structural constituent of chromatin [GO:0030527]
A0A162VCT1	UPI0007BB2ADE	Structural constituent of ribosome	cytosolic large ribosomal subunit [GO:0022625]; RNA binding [GO:0003723]; structural constituent of ribosome [GO:0003735]

#### 3.2.3 Comparison of *A. rabiei* EV‐Like Particle Proteome in the Presence/Absence of the Host

Among all the EV proteins detected in the PDB and PDB+Ch samples, ten proteins were significantly more abundant in PDB+Ch EVs compared to the PDB EVs (*p* value ≤ 0.05 was considered significant; Table 3), including two proteolytic enzymes (aminopeptidase, A0A162ZHB5; tripeptidyl‐peptidase II, A0A163ECM4), a RING‐type E3 ubiquitin transferase enzyme (A0A976RF86), a lyase enzyme (A0A163IXQ7), a redox‐active protein (thioredoxin, A0A163ETY3), and three uncharacterized proteins (A0A8H7MUX3, A0A8H7JHQ2, and A0A162WCM2) with identified homologous.

**TABLE 3:** *A. rabiei* EV proteins that showed significant variations in abundance depending on the presence (PDB+Ch) or absence (PDB) of the host.

Uniprot ID	Protein name	log2FC	*p* value	GO terms	More abundant in
A0A163LY48	Pectinesterase	2.33	0.020	extracellular region [GO:0005576]; aspartyl esterase activity [GO:0045330]; pectinesterase activity [GO:0030599]; cell wall modification [GO:0042545]; pectin catabolic process [GO:0045490]	PDB
A0A8H7JGR1	Endo‐polygalacturonase	0.73	0.039	polygalacturonase activity [GO:0004650]; cell wall organization [GO:0071555]; pectin catabolic process [GO:0045490]	PDB
A0A162XAW4	Pyridoxal 5'‐phosphate synthase (glutamine hydrolyzing)	1.05	0.050	pyridoxal 5'‐phosphate synthase (glutamine hydrolysing) activity [GO:0036381]; pyridoxal phosphate biosynthetic process [GO:0042823]	PDB
A0A163IVJ7	RNA helicase	−1.41	0.001	nucleus [GO:0005634]; ribonucleoprotein complex [GO:1990904]; ATP binding [GO:0005524]; helicase activity [GO:0004386]; hydrolase activity [GO:0016787]; nucleic acid binding [GO:0003676]; mRNA processing [GO:0006397]; RNA splicing [GO:0008380]	PDB+Ch
A0A163JDZ1	ATP binding	−1.52	0.004	cytoplasm [GO:0005737]; proteasome regulatory particle, base subcomplex [GO:0008540]; ATP binding [GO:0005524]; ATP hydrolysis activity [GO:0016887]; proteasome‐activating activity [GO:0036402]; protein catabolic process [GO:0030163]	PDB+Ch
A0A162WCM2	Uncharacterized protein	−2.89	0.012		PDB+Ch
A0A163IXQ7	Lyase	−1.52	0.014	extracellular region [GO:0005576]; pectate lyase activity [GO:0030570]; polysaccharide catabolic process [GO:0000272]	PDB+Ch
A0A162ZHB5	Aminopeptidase	−1.24	0.023	aminopeptidase activity [GO:0004177]; metal ion binding [GO:0046872]; metalloexopeptidase activity [GO:0008235]; proteolysis [GO:0006508]	PDB+Ch
A0A8H7MUX3	Uncharacterized protein	−2.87	0.026		PDB+Ch
A0A8H7JHQ2	Uncharacterized protein	−3.46	0.027		PDB+Ch
A0A976RF86	RING‐type E3 ubiquitin transferase	−0.83	0.030	ubiquitin‐dependent protein catabolic process [GO:0006511]; protein polyubiquitination [GO:0000209]; ERAD pathway [GO:0036503]; ubiquitin ligase complex [GO:0000151]; nucleus [GO:0005634] cytoplasm [GO:0005737]; ligase activity [GO:0016874]; ubiquitin‐ubiquitin ligase activity [GO:0034450]	PDB+Ch
A0A163ETY3	Thioredoxin	−2.71	0.041	protein‐disulfide reductase activity [GO:0015035]	PDB+Ch
A0A163ECM4	Tripeptidyl‐peptidase II	−1.94	0.044	extracellular space [GO:0005615]; metal ion binding [GO:0046872]; serine‐type endopeptidase activity [GO:0004252]; proteolysis [GO:0006508]	PDB+Ch

Also, three proteins were significantly more abundant in PDB EVs compared to the PDB+Ch EVs (*p* value ≤ 0.05 was considered significant; Table 3), including two cell wall‐degrading enzymes (pectinesterase, A0A163LY48; endo‐polygalacturonase, A0A8H7JGR1), and a pyridoxal 5'‐phosphate synthase protein (A0A162XAW4; Table 3).

In summary, the most enriched proteins detected with and without the host were membrane transporters (e.g., ATP‐binding cassette transporter, ATP hydrolysis), GTP binding and GTPases, proteins involved in translation (e.g., ribosomal subunits, RNA helicase, translational elongation factors), and proteins involved in pathogenic fungal infection processes (e.g., tricarboxylic acid cycle) (Figures 4 and 6, Tables S3 and S4). Additionally, proteins involved in cytoskeleton organization, protein folding, and fungal pathogenesis/virulence were identified (Figures 4 and 6, Tables S3 and S4).

Proteins enriched in the non‐host PDB group were related to DNA replication and transcription, amino acid metabolism and peptidase activities, cellular stress response, and cellular membrane components (Table S3). Meanwhile, those enriched in the presence of the host were related to oxidation‐reduction and cofactor metabolism, cell wall biosynthesis and components, lipid and isoprenoid metabolism, and nucleotide metabolism, ion transport and homeostasis, as well as cellular transport and vesicle trafficking (Table S4).

#### 3.2.4 Comparative Analysis of EV Proteomes Across Fungal Pathogens

**FIGURE 8:**
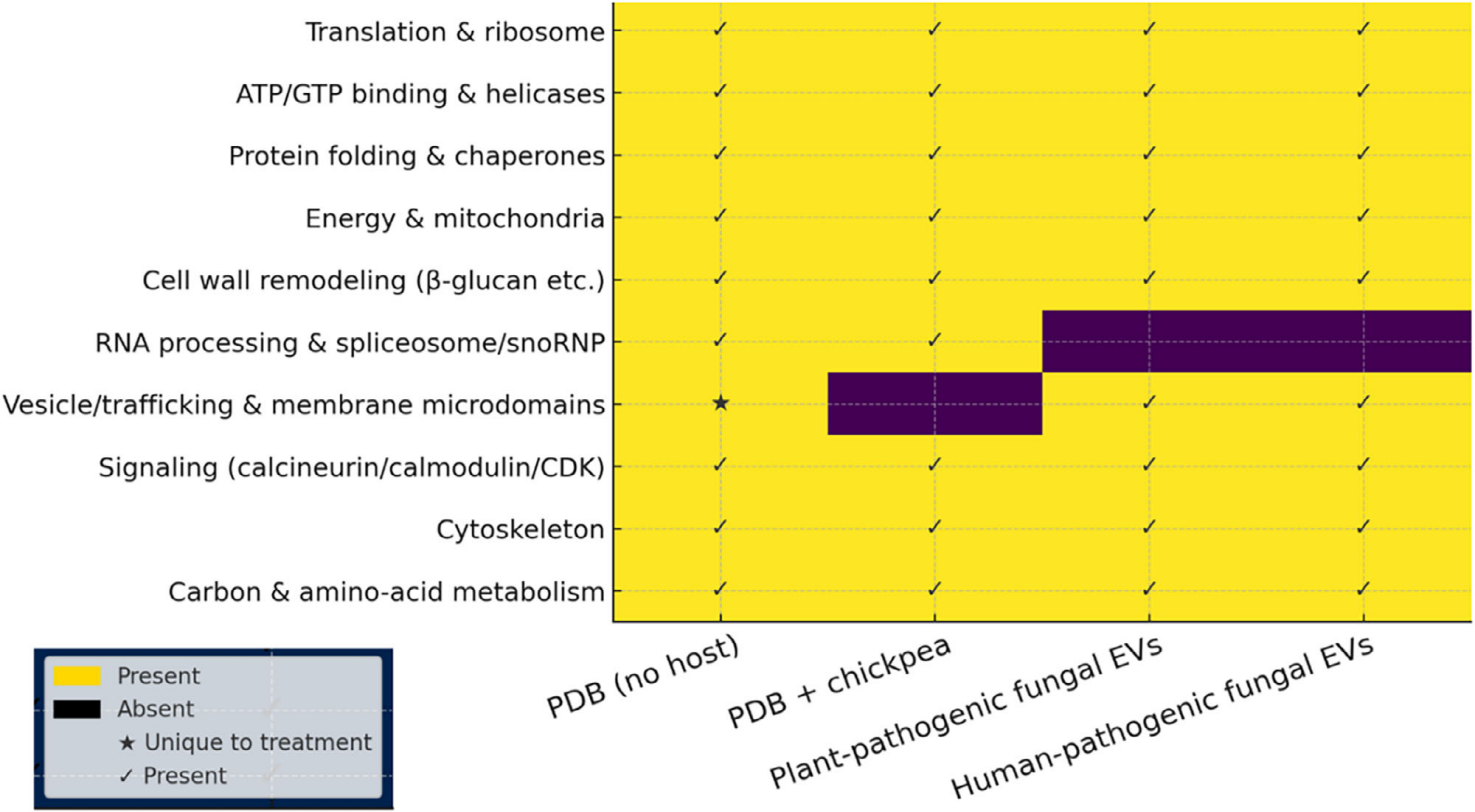
Comparative heatmap of functional GO categories in fungal EV proteomes. Heatmap showing enriched gene Ontology categories in EV proteomes from *Ascochyta rabiei* (PDB and PDB+chickpea) and representative plant‐ (*Fusarium oxysporum*, *Botrytis cinerea*, *Zymoseptoria tritici*) and human‐pathogenic fungi (*Candida albicans*, *Cryptococcus neoformans*, *Aspergillus fumigatus*). Ten functional groups were compared, including translation, ATP/GTP‐binding, protein folding, energy metabolism, cell wall remodeling, RNA processing, vesicle trafficking, signaling, cytoskeleton, and carbon/amino acid metabolism. Yellow cells in the heatmap indicated the presence of a functional category, black cells its absence, ✓ denoted detection across multiple datasets, and ★ denoted functional enrichment unique to one *A. rabiei* condition.

Comparison of the *A. rabiei* EV proteome with published fungal EV datasets revealed both conserved and condition‐specific functional signatures (Figure 8). Functional categories consistently detected across *A. rabiei*, plant‐pathogenic, and human‐pathogenic fungi included translation and ribosomal proteins, ATP/GTP‐binding proteins (helicases and GTPases), protein folding and chaperones, mitochondrial energy metabolism, cytoskeletal proteins, and signaling modules. These categories represent a conserved EV‐associated core, broadly shared across pathogenic fungi.

Condition‐specific protein enrichment was observed. In *A. rabiei*, RNA processing/spliceosome‐related proteins and vesicle trafficking or membrane microdomain‐associated proteins were enriched under host‐present (PDB+chickpea) conditions, while proteins linked to carbon and amino acid metabolism were predominantly enriched under host‐absent (PDB) conditions. The treatment‐specific enrichments indicate that host‐derived signals affect the selective incorporation of proteins into EVs. Notably, the enrichment of RNA‐ and trafficking‐related proteins in host‐present EVs aligns with reports from *Magnaporthe oryzae* [52] and *U. maydis* [16], where EVs contribute to the export of RNA‐binding proteins and vesicle‐associated factors during infection.

## 4 Discussion

This study reports for the first time that EV‐like particles are produced in *A. rabiei*, both in the presence and absence of the host. The *A. rabiei* EVs, ranging from 40 to 200 nm in diameter, were similar in size and morphology to EVs detected in other phytopathogenic fungi, including *F. oxysporum*, *F. graminearum*, *Z. tritici*, *A. infectoria*, and *C. higginsianum*, as was their cup‐shaped morphology [7, 14, 15, 17, 19]. Interestingly, this size range and morphology were also found in *Saccharomyces cerevisiae* [53]. However, larger vesicles (exceeding 400 nm in diameter) have been identified in some fungi, such as *B. cinerea* [11], and *Malassezia sympodialis* [54], but were not observed in this study, as the culture supernatant was filtered using 0.22 µm filters. This decision was made to ensure the integrity of the EV samples. Variations in size may result from different techniques employed for EV separation in different studies [55]. Additionally, since the supernatant from cultures of intact mycelia did not contain appreciable amounts of particles when examined by NTA and TEM, we believe these vesicles are genuine EVs rather than resulting from ruptured hyphal tips or damaged protoplasts.

The number of proteins identified in *A. rabiei* EVs, both in the presence (517 for PDB+Ch) and absence (276 for PDB) of the host, surpasses the numbers established for some of the other phytopathogenic fungi EVs, such as *F. oxysporum* f. sp*. cubense* (134 proteins) [13], and *P. capsici* (208 proteins) [5], *Z. tritici* (210 proteins) [15], and *A. infectoria* (20 proteins) [19]. However, some other phytopathogenic fungi have been found to produce EVs with a greater number of proteins, such as *B. cinerea* (617 proteins) [12] and *C. higginsianum* (over 700 proteins) [17]. Differences in the number of proteins detected in fungal EVs could be attributed to the type of mass spectrometry instruments, the databases utilized for peptide matching, the statistical rigor of mass spectrometry data analysis, and variation in EV isolation and processing methods.

Based on functional annotation, the protein cargo of *A. rabiei* EVs is predicted to consist mainly of enzymes involved in metabolism, oxidation/reduction, signaling, translation, and transport, along with ribosomal proteins, which were previously reported in other phytopathogenic fungi EVs, including *B. cinerea* [11, 12], *F. oxysporum* [7, 13], *F. graminearum* [14], *Z. tritici* [15], *C. higginsianum* [17], and *P. digitatum* [18]. Enrichment of oxidation‐reduction related proteins in *A. rabiei* EV proteome suggests the predicted role of these EV particles in response to oxidative stress, as reported in other fungi EVs, including *F. graminearum*, *F. oxysporum*, and *P. capsici* [5, 7, 14]. In addition, the *A. rabiei* EV‐like proteomes included several GTPases, specifically Ras and Rho GTPases, which regulate vesicle trafficking (Tables S1 and S2), and were previously reported in *B. cinerea* [11], *F. oxysporum* [7], *F. graminearum* [14], *C. higginsianum* [17], *P. digitatum* [18], and *P. capsici* [5]. Furthermore, proteins linked to the synthesis of secondary metabolites were also identified in *A. rabiei* and other phytopathogenic fungi EVs, such as propionate and methylcitrate. These metabolites represent putative factors predicted to contribute to pathogenicity or virulence [17]. For example, proteins associated with secondary metabolites have been detected in EVs isolated from *Z. tritici* [15]. Additionally, proteins encoded by previously recognized secondary metabolite biosynthetic gene clusters (BGCs) associated with the production of higginsianins (BGC16) were also discovered in *C. higginsianum* EVs [17].

Furthermore, several plasma membrane proteins and cell wall remodeling enzymes were also detected in *A. rabiei* EVs; these represent putative virulence‐associated factors, previously implicated in phytopathogenic fungi in host invasion, immune evasion, and fungal adaptability [6] (Table S5), which were previously reported to be present in other fungal EVs, including *F. oxysporum* [7, 13] and *F. graminearum* [14]. Membrane‐associated proteins were also found in the *A. rabiei* EV proteome, including SNAREs and GTPases, which have been previously suggested as markers for fungal EVs in *C. albicans* [49] and *C. higginsianum* [17]. The identification of SNARE proteins (Vesicular‐fusion protein SEC18, A0A163CAA6) and GTPases in *A. rabiei* EV proteome indicates that the proteome of *A. rabiei* EVs was successfully isolated and characterized, and suggests that EVs may represent unique vesicles rather than mere cellular debris. Although validation of these markers remains necessary, fungal EVs exhibit morphological similarities to EVs from other organisms and are isolated using protocols derived from established methodologies for mammalian EVs that are widely recognized and accepted.

To the best of our knowledge, this is the first study to investigate the production and cargo of phytopathogenic fungal EVs in relation to the presence or absence of the host in the culture medium. A comprehensive understanding of EVs produced by pathogens in the presence of the host facilitates the understanding of interkingdom communication involving EVs. Firstly, the production and size of the *A. rabiei* EV‐like particles varied significantly in the presence or absence of the host in the culture medium. Secondly, the protein cargo of *A. rabiei* EV‐like particles also changed based on the presence or absence of the host in the culture medium. The cargo of the EV proteome from various plant and human fungal pathogens, including *F. oxysporum* f. sp. *vasinfectum* [10] and *Histoplasma capsulatum* [56], has also been documented to vary according to the growth medium employed; however, they did not investigate the effect of host presence or absence in their experiments. After comparing the cargo and relative intensity of each protein identified in *A. rabiei* EVs, ten proteins were upregulated in the presence of the host (Table 3), including two proteolytic enzymes: aminopeptidase and tripeptidyl‐peptidase II, which play important roles in pathogenesis and induction of defence responses in plants [57]. For instance, in *Fusarium solani*, aminopeptidases have been identified as putative virulence factors, contributing to the pathogen's ability to infect and cause disease in host plants [58]. ​In another study reported by Olivieri et al. (2004), high extracellular proteolytic activity in *Spunta potato* tubers during *F. solani* infection establishes these proteolytic enzymes as crucial regulators of infection and as factors that prime immunity in potato as the host [59]. In addition, extracellular peptidases were reported as possible markers of ecological features of fungi [60]. The other detected protein that was found in higher levels in the presence of the host was a RING‐type E3 ubiquitin transferase enzyme that is pivotal in phytopathogenic fungi because it regulates the stability and turnover of key proteins, including virulence factors, stress response regulators, and effectors [61]. For instance, *M. oryzae*, which causes rice blast disease, ubiquitination (through E3 ubiquitin enzymes) plays important roles in growth, pathogenicity, stress response, and effector‐mediated plant‐pathogen interaction [29, 62]. Another detected enzyme was a pectate lyase, which plays a crucial roles in pathogenesis by degrading pectin, a major component of the plant cell wall, via a β‐elimination reaction [63]. The other protein was a thioredoxin, which is a small redox‐regulating protein that plays a crucial role in oxidative stress defence, fungal virulence, enzyme regulation, and cellular homeostasis in phytopathogenic fungi (Table 3) [64]. For instance, Ma et al. (2018) reported that thioredoxin‐ and glutaredoxin‐coding genes in *A. alternata*, were expressed in response to oxidative stress, and they are required for oxidative stress resistance, hyphal elongation, fungal growth, and virulence [64]. It is hypothesized that the increased abundance of these *A. rabiei* particle‐derived proteins in response to the host presence suggests a coordinated response involving stress adaptation, tissue degradation, nutrient acquisition, secondary metabolism regulation, and potential host interaction. The inclusion of EV‐related proteins in the presence of the host suggests that the pathogen might be actively modulating the host environment by transferring molecules that affect immune responses, alter plant defences, or facilitate pathogen entry and establishment in the host tissue. These findings highlight the functional importance of fungal EVs in the host‐pathogen interaction and provide insight into the molecular mechanisms underlying fungal adaptation in the presence of a host.

It is noteworthy that finding proteins with signal peptides, suggesting their secretion via EVs, an unconventional pathway for secretion, was also identified among the *A. rabiei* EV proteins. The number of these proteins with signal peptides varies among other phytopathogenic fungal EVs. For instance, only 6.7% of EV proteins from the *Z. tritici* exhibited a predicted signal peptide [15], whereas 12.5%–22% of EV proteins from *F. oxysporum* displayed a predicted signal peptide, contingent upon the growth medium utilized for the fungus [7, 14]. In another research, 4.9% of *C. higginsianum* low‐density vesicle proteins and 16.4% of high‐density vesicle proteins possess a projected signal peptide. In this research, 18.48% of *A. rabiei* PDB vesicle proteins and 13.37% of *A. rabiei* PDB+Ch vesicle proteins possess a projected signal peptide (Tables S1 and S2).

The comparative analysis demonstrated that fungal EVs share a conserved proteomic core, comprising translation machinery, energy metabolism, and stress‐associated proteins. This conservation across phylogenetically distant fungi, including both plant and human pathogens, supports the notion that EVs play fundamental roles in fungal physiology and intercellular communication. At the same time, *A. rabiei* exhibited host‐dependent modulation of its EV proteome. RNA‐processing and trafficking‐related proteins were preferentially enriched under host‐present conditions, whereas carbon and amino acid metabolic proteins were more prominent under host‐absent conditions. These findings suggest a functional shift in EV cargo toward host‐interactive processes during infection. Such adaptive remodeling has also been described in other plant pathogens and may reflect a mechanism by which necrotrophic fungi optimize EV‐mediated delivery of effectors and regulatory molecules in response to host‐derived signals.

Beyond the conserved functional categories, the *A. rabiei* EV proteome contained β‐1,3‐glucanosyltransferases and other cell wall–remodeling enzymes, enzymes associated with core metabolism and secondary metabolite biosynthesis, as well as several predicted effector proteins lacking classical secretion signals. Similar proteins have been reported in EVs from *B. cinerea* [65] and *F. oxysporum* [7] for CWDEs, in *Z. tritici* [15] and *C. neoformans* [50] for metabolic enzymes, and in *M. oryzae* [16] for effectors. These findings suggest that EV‐mediated delivery of virulence factors is a conserved strategy across fungal pathogens.

Finally, future research should concentrate on characterizing EVs under various growth circumstances, confirming fungal‐specific EV markers, and investigating their presence and function during in vivo chickpea infection, as the EV protein cargo in planta may substantially differ from that reported in vitro. The gene expression profiles of fungi in vivo and in vitro are different, and the culture conditions employed may not accurately replicate those encountered by the pathogen during chickpea infection. A comparative analysis of EVs derived from various fungal morphologies may yield additional insights; nonetheless, investigations of EVs extracted from the chickpea apoplast during *A. rabiei* infection in both susceptible and resistant cultivars will be essential to elucidate their role in pathogenesis. While technically challenging, recent studies in other plant–fungal systems have demonstrated that EVs can indeed be recovered from the apoplast, for example, in *Brassica napus* infected with *Leptosphaeria maculans* [66]. Moreover, fungal EVs are hypothesized to move through the apoplast and be internalized by host cells via clathrin‐mediated endocytosis or membrane fusion, thereby delivering protein and RNA cargo into recipient cells [12, 67].

The present study offers a proteomic analysis of *A. rabiei* EVs; however, direct evidence of their internalization by host cells was not evaluated. Subsequent research will concentrate on imaging and co‐localization experiments to elucidate the mechanisms of fungal EV internalization and trafficking during chickpea infection. These endeavors will be crucial in clarifying the function of EVs in fungal pathogenesis and host interactions.

## Author Contributions

M.G. designed the experiments, collected the data, performed the analysis of data, and wrote the manuscript. R.F. supervised the project, designed the experiments, reviewed and edited the manuscript. P.T.S., I.B., C.P., and M.J.A.S. designed the experiments, reviewed and edited the manuscript. A.J. processed the mass spectrometry samples and performed the statistical analysis of data. D.G.‐C., and I.B. performed the statistical analysis of mass spectrometry data. All authors read the manuscript and accepted co‐authorship. All authors have read and agreed to the published version of the manuscript.

## Conflicts of Interest

The authors declare no conflicts of interest.

## Supporting information




**Supporting File 1**: pmic70060‐sup‐0001‐TablesS1–S2.xlsx.

**Supporting File 2**: pmic70060‐sup‐0002‐TablesS3–S4.xls.

**Supporting File 3**: pmic70060‐sup‐0003‐TablesS5.xlsx.

## Data Availability

The mass spectrometry data supporting the findings of this study are openly available in the Zenodo repository under the dataset identifier DOI: https://doi.org/10.5281/zenodo.15743559.
